# Stem Cell Transplantation in Friedreich Ataxia: Cure for Leukemia but No Effect on Neurological Progression

**DOI:** 10.1002/acn3.70455

**Published:** 2026-07-13

**Authors:** Alexandra Gitman, Niyati Bhandari, Maria Castellaro, Kim Schadt, María Cancio, David R. Lynch

**Affiliations:** ^1^ Division of Neurology Children's Hospital of Philadelphia Philadelphia Pennsylvania USA; ^2^ Stem Cell Transplantation and Cellular Therapies, MSK Kids Memorial Sloan Kettering Cancer Center New York City New York USA

**Keywords:** ataxia, echocardiograph, leukemia, stem cell, transplant

## Abstract

Friedreich Ataxia (FRDA) is a neurodegenerative disorder of children and young adults associated with cardiomyopathy and other systemic complications. We report a 10‐year‐old girl who presented simultaneously with Acute Myelogenous Leukemia and FRDA who was successfully treated for her leukemia with allogeneic hematopoietic stem cell transplant. She experienced no major complications. After the transplant, her neurologic disease progressed similarly to other patients, but her blood frataxin levels returned to normal and cardiac hypertrophy decreased. This shows that FRDA patients can be treated with bone marrow transplantation, but such treatment alone has no direct effect on progression of neurologic disease.

## 1 Introduction

Friedreich's ataxia (FRDA) is a genetic, recessive neurodegenerative disease resulting from the loss of expression of frataxin protein [1, 2]. It affects the central nervous system (CNS), muscle (including the heart), pancreatic islet cells, and other regions, leading to optic neuropathy, ataxia, spasticity, weakness, scoliosis, hypertrophic cardiomyopathy, and diabetes [1, 2]. FRDA is caused by biallelic GAA expansions in the *FXN* gene in 96% of people, leading to a deficiency of frataxin [1, 2].

While many therapies in development for FRDA aim to mitigate downstream consequences of frataxin deficiency, others seek to restore frataxin expression directly using approaches such as gene therapy, protein replacement, and epigenetic activation [2]. Current gene‐therapy strategies primarily rely on in vivo delivery of the FXN gene using adeno‐associated viral vectors to increase frataxin expression in affected tissues, particularly the heart and nervous system. Although ex vivo correction of the GAA repeat expansion has been explored in experimental settings, it is not currently an approved clinical treatment for FRDA [3]. Conceptually, the cells of the monocyte lineage could enter the brain, the main site of active degeneration in FRDA, and transfer frataxin to deficient cells. This has been proposed based on potential exchange of material between migrating cells and the endogenous cells of the brain. Such an approach has potential advantages but also possible risks, and the biological mechanisms supporting its utilization are not fully defined. Here, we report a patient who presented simultaneously with FRDA and acute myeloid leukemia, the latter of which was treated with stem cell transplantation from an allogeneic donor. Her course may provide relevant information on the safety and potential benefit of stem cell‐based approaches for frataxin replacement in the context of progressive neurological impairment and potential chemotherapy induced exacerbation of cardiomyopathy.

## 2 Results

A 10‐year‐3‐month girl presented with chest pain and tachycardia and was found to have thyrotoxicosis requiring admission to the intensive care unit (ICU). During hospitalization, she was noted to have persistent cytopenias. A bone marrow aspirate revealed high risk Acute Myeloid Leukemia (AML t(6;9)) without CNS involvement. Simultaneously, she was noted to have a slightly wide based gait with externally rotated legs, ataxia, minimal tremors isolated to her fingers, a small decrease in vibratory sensation, and absence of deep tendon reflexes. Brain MRI scan was normal. Genetic testing revealed biallelic expanded GAA repeats in FXN of 699 and 1066, confirming the diagnosis of FRDA. An echocardiograph identified moderate left ventricular hypertrophy.

AML treatment consisted of induction chemotherapy with dose adjusted ADE (cytarabine, daunorubicin, and etoposide). The patient failed to achieve remission and was transitioned to TVTC (topotecan, vinorelbine, thiotepa, clofarabine), attaining minimal residual disease (MRD) negativity after two cycles. Subsequently, she underwent an allogeneic hematopoietic stem cell transplant from an 11/12 HLA‐matched unrelated male donor, using CD34+ selected peripheral blood stem cells (PBSCT). Cardiac MRI prior to transplant showed normal left ventricular size and function (LVEF 74%), borderline septal wall thickening (1.1 cm), and no clear evidence of myocardial infarction or fibrosis, though image quality was suboptimal. Right ventricular size and function were normal (RVEF 62%). Her conditioning regimen consisted of rabbit anti‐thymocyte globulin (ATG), clofarabine, melphalan, and thiotepa. No major unexpected complications occurred, and she had no graft vs. host disease. The patient went into remission, with no reappearance of AML over the next 10 years.

**FIGURE 1:**
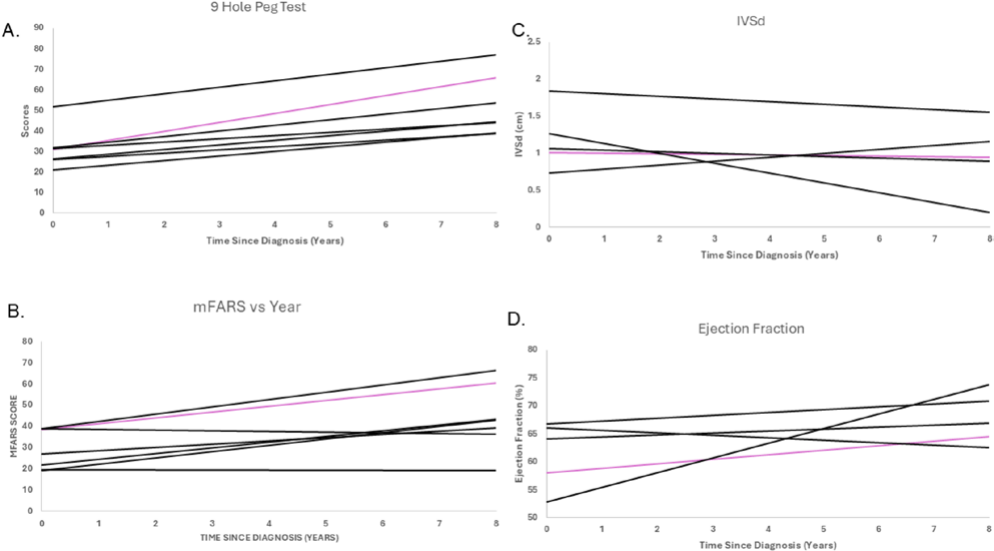
Change in FRDA outcome measures after PBSCT. Graph of change in the FRDA outcome measures 9‐hole peg test (9HPT) (A), mFARS (B), Intraventricular septal diameter (IVSd) (C), and cardiac ejection fraction (D) over time. The patient reported here shown in colored purple line, while other FRDA patients presenting at age 9 are shown in black. There was no consistent change in neurologic measures from expected progression, but cardiac measures showed little change over the time since transplant. The six comparator patients had repeat lengths/age of onset of: 700/1333, 10 years; 833/1033, 10 years; 700/1066, 8 years; 699/1066, 8 years; 599/1066, 9 years; 599/1066, 9 years. All compare favorably with the patients' values of 699/1066, 10 years. All have been followed for similar lengths of time with similar testing over that time (although only 4 had cardiac follow up). The selection of the comparators provides a context for showing that the patient continues to progress at a similar rate to others, though small differences may occur as the variability in progression of FRDA requires larger sample sizes for sufficient power to distinguish small differences [4, 6].

Over the next decade following transplant, she slowly progressed neurologically with mildly affected speech and worsening gait and arm function. Reflexes were unchanged and peripheral neuropathic components showed no reversal. Additionally, she developed diabetes and sleep apnea but did not require surgery for scoliosis. Clinical examinations matched progressive increases in both ataxia and spasticity, as quantified with modified Friedreich Ataxia Rating Scale scores (increase of 20 points over 10 years) and 9‐hole peg test scores (doubling over 10 years). The degree of progression was similar to six other FRDA patients presenting at the age of 8–10 years with similar GAA repeat lengths to the same institution within 5 years of this subject (Figure 1) [4]. At present, she uses a wheelchair and does not ambulate independently. Interestingly, while she experienced neurological deterioration, her cardiac hypertrophy remained unchanged, such that her echocardiograph 10 years after transplant was read as normal. Genetic testing in blood returned to normal (GAA repeat lengths of 10 and 16), as did blood frataxin levels (Frataxin M = 4.79 ng/mL; Frataxin E = 9.25 ng/mL) [5].

## 3 Discussion

Stem cell transplantation (SCT) is indicated in patients with high‐risk t(6;9) AML due to high risk of relapse with standard chemotherapy alone. Although allogeneic SCT did not reverse or stabilize neurologic disease in FRDA, the present case provides evidence that affected FRDA individuals can survive allogeneic SCT, similar to their ability to survive other procedures such as heart and liver transplantation [7, 8]. This report illustrates that a successful allogeneic transplant is achievable in FRDA as a treatment for hematologic malignancies and other disorders.

Unfortunately, while introduction of stem cell replacement normalized the genetic FRDA mutation and frataxin levels in blood, this approach did not have a major effect on neurologic progression in FRDA. Although peripheral neuropathy is difficult to evaluate in FRDA due to its developmental loss, this patient progressed neurologically (on tests directed at ataxia) at a similar rate to FRDA patients of comparable age and age of onset, affecting both the upper and lower extremities, and eventually leading to the patient being wheelchair bound at roughly the expected time. The failure of any response in the central nervous system likely reflects a combination of factors. First, the blood brain barrier prevents most donor derived cells from entering CNS parenchyma. Traditional SCT does not achieve microglial monocyte depletion; monocyte depletion beyond allogeneic SCT may be required to provide benefit [9]. Frataxin is an intracellular, mitochondrial protein; therefore, donor cells do not typically participate in cross‐correction. In addition, FRDA has significant developmental components; replacement of frataxin later than the developmental period may limit its benefit [10, 11]. Finally, the treatments needed for AML (which may exacerbate neuropathy) may blunt benefit from SCT, and secondary diabetes from FRDA or other causes may interfere with beneficial effects. Thus, frataxin deficiency in the nervous system may remain despite engraftment and full donor chimerism. Therefore, in this patient, ataxia and neuropathy are expected to progress despite successful hematopoietic replacement.

Interestingly, some cardiac features of FRDA in this patient stabilized over the 10 years following treatment. When comparing ejection fraction and IVD_s_ to other patients with similar ages of onset and GAA repeat length, this patient's values stabilized leading to an eventual absence of hypertrophy, though this was also noted in some of the comparator subjects. While such changes may suggest meaningful benefit, hypertrophy in FRDA is modestly predictive of eventual outcome and decreases in wall thicknesses alone can occur without long‐term benefit [12]. Changes in ejection fraction are typically late phenomena in FRDA; thus, its stability over 10 years is notable but not definitive.

When her genetic test for FRDA was repeated 10 years after transplant, GAA repeat lengths were normal, and frataxin levels returned to the control range. This reflects the transplanted stem cells she received, which differentiate into genetically unaffected cells of the donor cells producing normal frataxin protein levels. As FRDA is not known to increase cancer risk, the presence of AML and FRDA in this patient is likely to be coincidental [13] but contributes greatly to treatment complexity given chemotherapy sensitivity, cardiac risk and neurotoxicity risk with some chemotherapy agents. Still, the successful treatment in this patient illustrates the opportunity for use of SCT in future patients with FRDA and various forms of cancer. In addition, the relative safety of the procedure and the possible cardiac stabilization here suggests that SCT could provide beneficial effects if used in a context designed to benefit CNS function. Still, the differences between the clinical use of SCT here and the paradigms needed for CNS frataxin restoration prohibit the use of the present observations as evidence for potential benefit in the CNS.

## Author Contributions

Alexandra Gitman and Niyati Bhandari wrote the initial draft and accumulated data. All authors provided critical editing and revising.

## Funding

This work was supported by Friedreich's Ataxia Research Alliance.

## Conflicts of Interest

The authors declare no conflicts of interest.

## Data Availability

The data that support the findings of this study are available on request from the corresponding author. The data are not publicly available due to privacy or ethical restrictions.
